# The influence of water in silicate melt on aluminium excess in plagioclase as a potential hygrometer

**DOI:** 10.1038/s41598-018-29178-z

**Published:** 2018-08-20

**Authors:** Alina M. Fiedrich, Lukas H. J. Martin, Julian-C. Storck, Peter Ulmer, Christoph A. Heinrich, Olivier Bachmann

**Affiliations:** Institute for Geochemistry and Petrology, Department of Earth Sciences, Zurich, 8092 Switzerland

## Abstract

Measuring water contents of magmas is fundamental to resolving a number of geological questions, such as the mechanisms of silicic magma evolution, the triggering of volcanic eruptions, and the formation of porphyry copper deposits. This study focuses on the correlation between apparent deviations from stoichiometry of plagioclase crystals and high water concentration in the magmatic melt from which they grew. We considered this relationship as a potential geo-hygrometer (water activity indicator). To test and potentially calibrate this new technique, a range of natural and experimental plagioclase crystals were analysed, with particular care taken to identify and avoid analytical bias and artefacts. In contrast to recently published material, we found no systematic aluminium excess in plagioclase, irrespective of the water concentration of the silicate melt it crystallised from. This suggests that aluminium excess in plagioclase cannot serve as a geo-hygrometer. The high likelihood of misinterpreting analytical artefacts (due to alkali migration and imprecise standardisation) as small deviations from stoichiometry, also requires its application as a mineral exploration tool to be treated with caution.

## Introduction

## Importance and Estimation of Pre-Eruptive Water Concentrations in Magmas

Determination of pre-eruptive water contents of magmas is key to understanding the evolution of magmas and subsequent implications for the formation of magmatic-hydrothermal ore deposits. The pre-eruptive concentration of water dissolved in the melt and the process of magma degassing influence the crystallising mineral assemblage, thereby controlling geochemical trends and affecting the eruptive style (e.g.^1–4^). In particular, dissolved water lowers solidus temperatures of rocks and therefore promotes melting^5,6^. Moreover, the initial concentration and subsequent enrichment of water and other volatile components in the melt critically control the formation of a free volatile phase, which can give rise to magmatic-hydrothermal ore deposits, such as porphyry mineralisations^7^.

At present, pre-eruptive water concentrations can be estimated through phase equilibria (typically involving geochemical analysis of minerals and melt; e.g.^8–18^), or by measuring melt inclusions or fresh, undegassed glass by various methods, including secondary ion mass spectrometry (SIMS), Fourier-transform infrared spectroscopy (FTIR), or Raman spectroscopy (e.g.^19–21^). Recently, H-compounds were measured directly in nominally anhydrous minerals^22^), including measurements of plagioclase^23–27^, and the use of structural-geochemical proxies for H as geo-hygrometers^28^. However, these methods either rely on experimental studies, require complicated measurement procedures, suffer from restricted applicability, or pertain to analytes that may not preserve their original compositions (e.g. due to H diffusion). Therefore, the petrological community is continually searching for alternative geo-hygrometers.

Here, the possibility to use excess aluminium in plagioclase (Al*) as a geo-hygrometer is explored. Excess aluminium in plagioclase is defined as Al* = ((Al/(Ca + Na + K) − 1)/X _*An*_) > 1 (in atoms per formula unit, apfu; An = anorthite content)^29^ and denotes the incorporation of more Al than given by the ideal stoichiometry. It appears to occur dominantly in plagioclase from mineralised calc-alkaline plutons and was hypothesised to be related to a high partial pressure of water (P _*H*2*O*_) in the melt^29^. Geochemical correlations support coupled substitution of AlAl_3_SiO_8_ and []Si_4_O_8_ and occupation of the generated vacancy by H_2_O as incorporation mechanism for water and Al into plagioclase^29,30^. However, studies applying Fourier-transform infrared spectroscopy (FTIR) showed that igneous plagioclase typically only contains OH, not H_2_O, although lattice positions of H incorporation could not always be identified (^22^ and references therein). Moreover, no correlation between H content and feldspar composition or geodynamic setting was found (summary in^31^, their Table 1), thereby supporting fast diffusion of H.

A hygrometer based on Al* would potentially bear the advantages of: (a) wide applicability due to the ubiquitous presence of plagioclase in most igneous rocks, (b) ease of measurement with standard-based wavelength dispersive X-ray spectrometry (WDS) attached to an electron-probe micro-analyser (EPMA), (c) good preservation potential due to slow diffusion of Al, Ca, and Na compared to H (e.g.^32–35^), and (d) potential to resolve changing concentrations recorded in mineral zoning. Therefore, the scope of this study is to test, if the incorporation of Al* in plagioclase can be developed into a geo-hygrometer, using both natural samples from a range of environments and experimentally-grown plagioclase crystals precipitated from melts with contrasting water contents. In the course of this evaluation, care was taken to minimise potential analytical artefacts that could lead to erroneous interpretations.

## Results

### Sample selection

Plagioclase crystals from a wide variety of samples (see electronic supplementary material) were selected for analysis with EPMA with the aim to: (a) reproduce the dichotomy in Al* between barren (samples without any concrete evidence for an associated porphyry copper deposit at depth) and mineralised lithologies as presented in^29^, (b) study the effect of P_*H*2*O*_ in the magma on Al* in plagioclase, and (c) explain other factors - geological-geochemical and analytical - influencing the measured Al* in plagioclase. Unaltered, natural plagioclase crystals can be found in barren magmatic rocks from both plate margin and intraplate environments, but also in some rocks related to porphyry copper deposits (electronic supplementary material). If the hypothesis of discernible Al* in mineralised and barren rocks as postulated in^29^ (their Fig. 1) is true, the mineralised sample group should exhibit more pronounced Al* than the barren one. The difference in Al* should be particularly pronounced between mineralised deposits and dry magmas from hotspots if P _*H*2*O*_ indeed controls the Al* in plagioclase. As an additional test, plagioclase crystals were produced experimentally from wet and dry melts in this study to further test the correlation between Al* and dissolved water concentration in the melt under controlled conditions.

### Mineral chemistry of natural plagioclase from different geological settings

**Figure 1:**
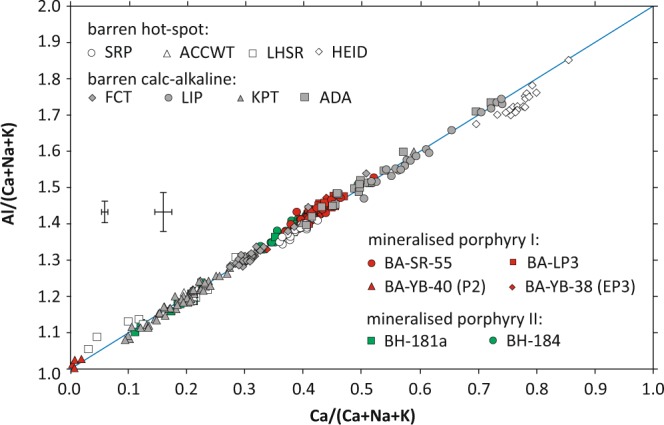
Compositional data of natural plagioclase obtained by EPMA. Grey symbols correspond to barren calc-alkaline, hollow symbols to hotspot-related, and red and green symbols to porphyry copper-related samples [FTC = Fish Canyon Tuff^42^; LIP = Lipari^43^; ADA = Southern Adamello^44^; KPT = Kos Plateau Tuff^45^; SRP = Central Snake River Plain^46^; ACCWT and LHSR = Walcott Tuff and Lidy Rhyolite from Heise^47^; HEID = Heidarspordur^48^; BA = Bajo de la Alumbrera^49,50^; BH = Batu Hijau^51,52^]. The left error bars corresponds to the average 2 *σ* uncertainty, the right error bars to the maximum 2 *σ* uncertainty (measurements on standards excluded). The blue line is referred to as stoichiometry line in the text.

From the analysis of 299 data points on plagioclase crystals from 14 natural samples (Fig. 1), it can be shown that: (a) Plagioclase compositions from different samples largely overlap in Al* (at similar anorthite content) and are narrowly distributed on both sides of the stoichiometry line, irrespective of the presumed water content in the magma. The only exception is plagioclase from a basaltic sample from Iceland (HEID), which plots mostly below the stoichiometry line. In agreement with the findings of Williamson *et al*.^29^, this may be related to its relatively high Fe content (>0.02 Fe (apfu)). Furthermore, no systematic variations in Al* in variably mineralised porphyry sample sets from Batu Hijau (samples BH-181a and BH-184) and Bajo de la Alumbrera (samples BA-SR-55, BA-LP3, BA-YB-38 EP3 and BA-YB-40 P2) could be observed. In addition, we note that partial albitisation of sample BH-181a is reflected in bimodal anorthite contents of plagioclase. Despite the fact that altered zones (quartz-magnetite and potassic) of plagioclase within the excitation volume could not be avoided in mineralised porphyry samples BA-YB-38 and BA-YB-40, these samples do not show significant deviation from the general trend. This makes the possibility of the bimodal distribution of Al* as found by^29^ being an artefact of microclay alteration unlikely.

With respect to analytical conditions, data treatment, and potential artificially-induced errors, it has been observed that: (a) inclusion of cations other than Ca, Na, and K at the M site, such as Ba and Sr, does not change the positions of most data sets visibly. (b) Analysis with normal beam current (ca. 20 nA) is necessary to yield sufficient intensities and uncertainties low enough to observe small differences in Al*. Larger uncertainties, on the other hand, result from reduced beam current for focused analyses and are sufficient only to resolve large differences in Al*. (c) A correction based on repeated analyses of the albite and anorthite standards as unknowns was necessary and resulted in all data to plot close to the stoichiometry line.

### Synthetic plagioclase crystallised from melts of contrasting water concentrations

**Figure 2:**
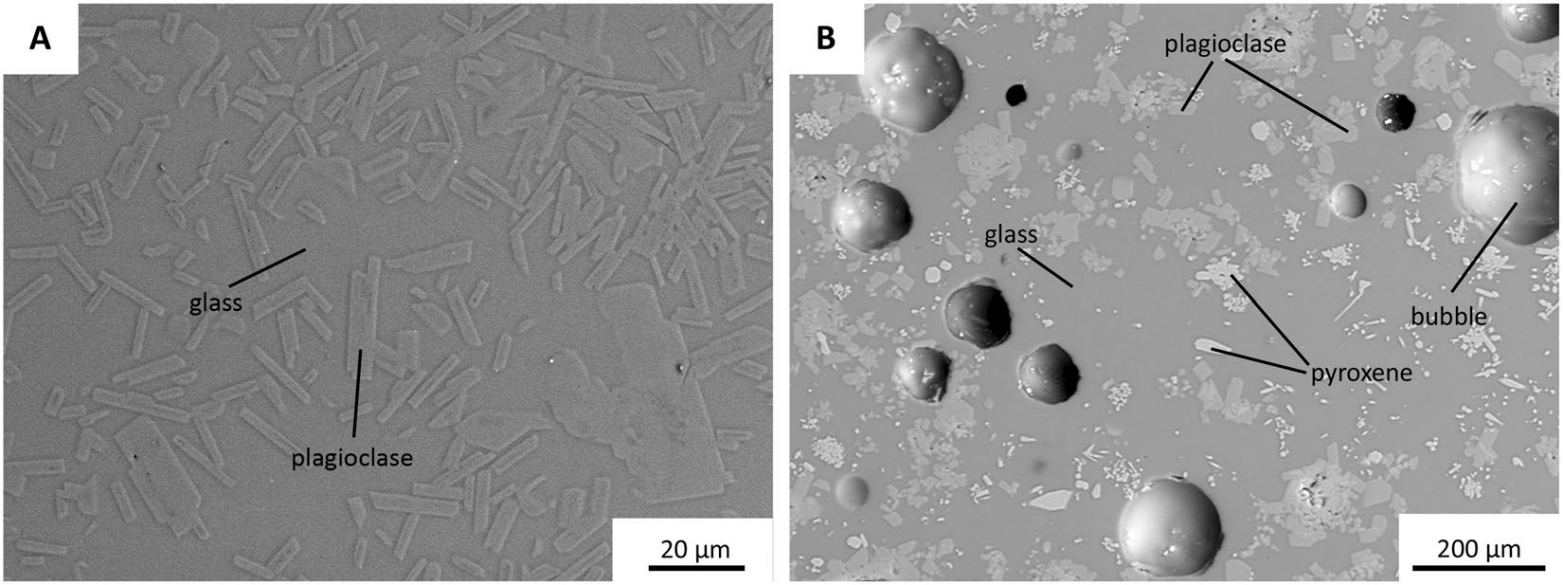
Back-scatter electron images of experimental samples. (**A**) Experiment E – high-temperature, water-poor: lath-shaped plagioclase crystals within glassy matrix. (**B**) Experiment D – low-temperature, water-rich: stubby columnar plagioclase (medium grey), short prismatic clinopyroxene (diopside, light grey), and bubbles within glassy matrix (dark grey).

The main differences between the experimental runs with wet and dry melts are the following: In the high-temperature, water-poor experiments, plagioclase formed crystals of high aspect ratio and was the only mineral that crystallised. In experiment C (1240 °C), single crystals were ca. 150 *μ*m long with hollow interiors (occupied by glass) and occurred only in the upper part of the capsule. In experiment E (1190 °C), plagioclase crystals rarely exceeded ca. 30  *μ*m length, were homogeneously distributed throughout the capsule, and occurred in random orientations (Fig. 2a). The resulting total crystallinity was ca. 50 area%.

In the low-temperature, water-rich experiment (experiment D, 925 °C), plagioclase co-crystallised with acicular to short-prismatic clinopyroxene (Fig. 2b), typically in the form of clusters. Plagioclase was more stubby in shape than in hot, dry experiments, and commonly up to 25 *μ*m long, although a few crystals reached up to 60 *μ*m length. Bubbles of variable size were distributed throughout the experimental capsule and cover ca. 20 area%. The overall crystallinity of this experiment was less than 50 area%.

**Figure 3:**
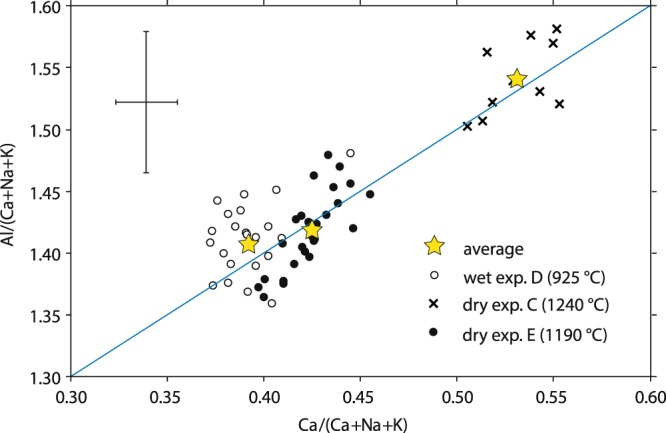
Compositional data of experimental plagioclase obtained by EPMA. Analyses of synthetic plagioclase do not show any analytically significant distinction between plagioclase from water-rich versus water-poor melts. Focused analyses with low beam current lead to a relatively high average uncertainty (black cross); crosses, hollow circles, and filled circles correspond to plagioclase compositions of experiments C, D, and E, respectively; yellow stars represent the average compositions for each experiment.

Plagioclase of all experiments was of similar, intermediate anorthite content (Fig. 3; average compositions in Table 1). Most plagioclase crystals were fairly homogeneous according to quantitative analyses and lack of SEM-BSE contrast. In the low-temperature, water-rich experiment (experiment D), however, plagioclase crystals in the core of clusters were of higher anorthite content. Iron oxide and K_2_O concentrations in plagioclase from all experiments were commonly <0.1 wt% and <0.05 wt%, respectively, i.e. below detection limit. Clinopyroxene and glass were homogeneous throughout the experimental capsule.

**Table 1:** Representative (average) analyses of experimental plagioclase (EPMA), pyroxene (SEM-EDS), and glass (SEM-EDS), in wt%; All EPMA data are listed in Appendix A. The difference of the totals of the glass analyses to 100 wt% can be used to approximate the water concentration of the glass.

	SiO_2_	Na_2_O	CaO	K_2_O	FeO	Al_2_O_3_	TiO_2_	MnO	MgO	Total
plagioclase - exp. C	54.34	5.25	10.79	d.l.	d.l.	28.40	0.01	0.03	0.09	98.94
plagioclase - exp. D	57.55	6.91	7.99	d.l.	d.l.	26.18	0.01	0.02	0.04	98.75
plagioclase - exp. E	57.34	6.46	8.68	d.l.	d.l.	26.31	0.02	0.02	0.15	99.03
pyroxene - exp. D	55.1	0.3	25.5	0.0	0.4	1.3	0.0	—	17.2	99.9
glass - exp. C	61.3	7.1	7.7	0.0	0.0	20.5	0.0	—	1.4	98.0
glass - exp. D	60.6	7.8	2.9	0.0	0.1	17.7	0.0	—	0.4	89.6
glass - exp. E	62.9	7.6	7.0	0.0	0.1	17.9	0.0	—	2.2	97.7

These values coincide well with tentative analyses using attenuated total reflection FTIR with a calibration for andesitic glass (yielding ca. 10 wt% total water for experiment D and <1 wt% total water in experiments C and E). Small amounts of K_2_O and FeO (d.l. = below detection limit, <0.1 wt% for FeO and <0.05 wt% for K_2_O) in the analytes are likely a result of minor contaminations of the starting powders.

Our results from experimentally crystallised plagioclase support the findings based on natural plagioclase: Through analysis of both natural and synthetic plagioclase from dry versus wet melts, large differences in Al* in plagioclase as presented in^29^ (their Fig. 1a) could not be reproduced.

### Sodium diffusion in plagioclase during analysis as the main mechanism to explain excess aluminium

**Figure 4:**
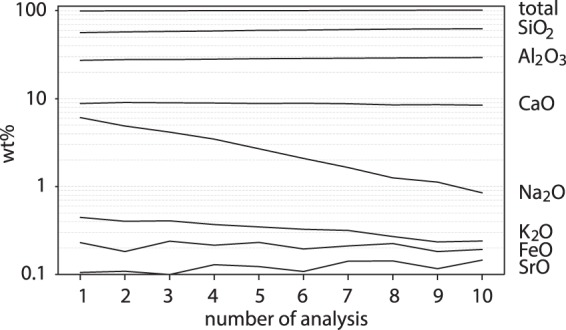
Repeated analysis (ca. 3 min each analysis) by EPMA-WDS of the same spot on plagioclase from sample BA-YB-38 EP3 to observe beam damage due to focused electron beam at 20 nA beam current.

Small differences in the composition of plagioclase can only be resolved using homogeneous and geochemically well constrained standards that match the matrix of the analysed mineral (e.g.^36^). Here, well-characterised end-member compositions of feldspars with nearly perfect recalculated stoichiometry assure that no analytical effect from false standard compositions can affect the results. In addition, instrumental and lab environment conditions (including room temperature, emission current, focal point, etc.) must be stable during an analytical session. In albite- or orthoclase-rich feldspars, alkali element migration under the electron beam of the EPMA is a prominent cause of analytical deviation from the actual composition, as the following example of repeated analysis with focused beam and 20 nA beam current shows (Fig. 4): Elements such as Ca and Fe stay relatively unaffected (around 8.8 ± 0.2 wt% CaO and 0.2 ± 0.02 wt% FeO) during repeated measurement with small beam diameter at high beam current at the EPMA. However, accumulated counts of Na and thus apparent Na_2_O concentrations markedly decrease (from 6.1 to 0.9 wt%) while apparent SiO_2_and Al_2_O_3_ concentrations increase (from 56.5 to 62.5 wt% and from 27.3 to 29.4 wt%, respectively). Moreover, the plagioclase composition did not recover even after several minutes without irradiation. Consequently, (a) the totals of the analyses, which are often used as an indicator of analysis quality, only increase slightly (from 99.6 to 101.7 wt%), (b) calculated apfu (based on 8 oxygens) of plagioclase decrease (from 5.00 to 4.63), (c) measured anorthite contents in plagioclase are increased artificially (here from ca. An _43_ to An _82_), and (d) data are shifted away from the stoichiometry line into the “fertile” field in the diagram Ca/(Ca + Na + K) vs. Al/(Ca + Na + K). This extreme example implies that repeat analyses and too long analysis times with a small beam diameter and high beam current can lead to small deviations from the stoichiometry line that may then be misinterpreted as Al*.

**Figure 5:**
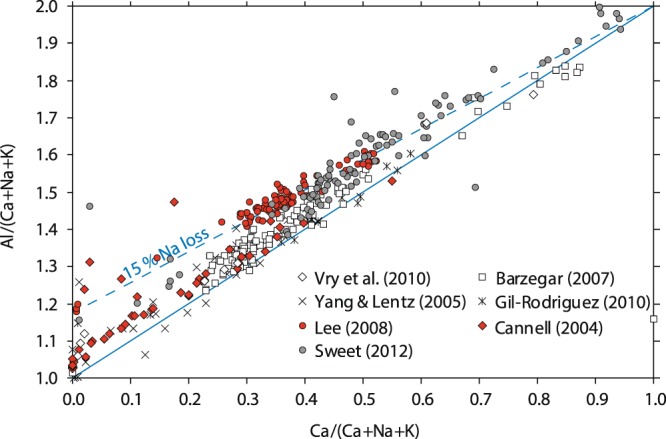
Some examples of plagioclase analyses from mineralised plutons presented in^29^, their Fig. 1. Full coverage of all datasets was not possible here because some datasets were inaccessible. The solid blue line represents stoichiometrically ideal plagioclase. The dashed blue line corresponds to 15% Na loss, calculated as: Na(apfu) = (1 − X_*An*_) * 0.85. Datasets as indicated in the legend from^53–59^.

We suspect that element diffusion may have occurred in the literature database presented by Williamson *et al*.^29^, their figure 1 (Fig. 5). In particular, the dataset highlighted in red, which shows apparently high Al*, follows a trend that corresponds well to ca. 15% Na loss (dashed line). Other datasets appear systematically offset as well, but follow trends (sub-)parallel to the stoichiometry line. This, in turn, might be an artefact related to the standardisation.

## Discussion

The study of both natural and synthetic samples indicates that Al* in plagioclase is not suitable as a geo-hygrometer due to analytical limitations. In particular, differences in Al* in plagioclase are too small to be resolved by EPMA-WDS routinely. Furthermore, the aluminium excess versus deficiency in plagioclase as described in^29^ is unlikely to be related to P_*H*2*O*_ in the melt because no differences between plagioclase compositions in samples from dry or wet melts were found. Independent of its incorporation mechanism, Al* in plagioclase does not appear to be a robust means to explore for mineralisation, as the mineralised samples studied here do not show any Al* variations beyond uncertainty. Seemingly large differences of Al* in plagioclase, as observed in literature compilations^29^, may instead be analytical artefacts due to inappropriate standardisation or Na loss during EPMA analysis. Hence, the correlation between mineralisation and Al* in plagioclase remains uncertain, and other factors that influence the mineral composition need to be studied.

## Methods

### Piston cylinder experiments

Plagioclase was crystallised from synthetic melts in the system diopside (Di) - albite (Ab) - An ± water^37^. The Di component was included for depolimerisation of the melt and to promote growth of larger crystals. Other components such as Fe and K were avoided, as they potentially disturb the incorporation of Al* in plagioclase^29^. Water concentrations were initially high in one experiment, intended to impose a significantly larger amount of dissolved water in the melt compared to the dry experiment. Starting compositions (Table 2) were tentatively calculated with Rhyolite-MELTS^38,39^. Experiments were then repeated with slightly modified temperature and bulk composition to reach (a) an intermediate anorthite content representative of many natural plagioclase compositions and (b) an intermediate crystallinity for sufficient material to analyse and sufficient interstitial melt to allow equilibration. Starting compositions were mixed from SiO_2_, *γ*-Al_2_O_3_, Al(OH)_3_, MgO, CaSiO_3_, and Na_2_SiO_3_ powders, which were dried in a furnace overnight. Starting materials for water-free runs were fused into glass in a Pt crucible in a 1 atm furnace at 1350 °C for 15 min to avoid uptake of water. Pt capsules were used for dry, higher temperature experiments (experiments C, E), and Au capsules were used for the water-bearing, lower temperature experiment (experiment D) to minimise loss of water (e.g.^40^). The outer capsule diameter was 4 mm, and the capsule length was around 10 mm. Experimental runs were conducted using a 14 mm bore, Boyd-England type (end-loaded) piston cylinder apparatus at the Department of Earth Sciences, ETH Zurich. The experimental assembly consisted of talc-pyrex-glass-graphite-MgO-parts. Temperature was controlled with a Pt_94_Rh_6_-Pt_70_Rh_30_ (type B) thermocouple. A friction correction of 10% to the nominal pressure but no pressure correction to the thermocouple electromotive force were applied. Pressure was 0.5 GPa, reflecting upper to mid-crustal conditions, enhancing crystal growth compared to lower pressures, and allowing stable pressure control with the piston cylinder apparatus. Heating rate was 60–80°/min. Pressure and temperature were held constant for 2–3 days before quenching. Quenching was performed by switching off the power, resulting in an initial quench rate >200 °C/s. A pressure uncertainty of ±300–500 bar is expected. Oxygen fugacity was not controlled (although the use of MgO parts typically results in rather oxidising conditions), as the experiment was free of Fe and S.

**Table 2:** Starting compositions (anhydrous, normalised to 100%; except for H_2_O), temperatures (T), and run durations (t) applied in piston cylinder experiments.

	Experiment C	Experiment D	Experiment E
An (mol%)	25	20	25
Ab (mol%)	65	70	65
Di (mol%)	10	10	10
SiO_2_ (wt%)	60.9	62.2	60.9
Al_2_O_3_ (wt%)	22.4	21.5	22.4
MgO (wt%)	1.5	1.6	1.5
CaO (wt%)	7.5	6.5	7.5
Na_2_O (wt%)	7.7	8.3	7.7
H_2_O (wt%)	—	5	—
capsule	Pt	Au	Pt
glass	yes	no	yes
T (°C)	1240	925	1190
t (h)	70	72	48

### Quantitative analysis

Plagioclase was analysed using a JEOL JXA-8200 electron probe micro-analyser (EPMA), equipped with five wavelength-dispersive X-ray spectrometers at the Department of Earth Sciences, ETH Zurich. Samples were polished and coated with a ca. 20 nm thick carbon layer to avoid heating of the sample under the electron beam and charging effects. For the analysis of all elements the K *α*-lines were used, except Sr and Ba, for which the L *α*-line was used. Standards used for calibration of the main elements in plagioclase, Si, Al, Na, and Ca, were synthetic anorthite (43.45 wt% SiO_2_, 36.46 wt% Al_2_O_3_, 0.29 wt% Na_2_O, 0.06 wt% K_2_O, 19.57 wt% CaO, 0.09 wt% FeO, 99.92 wt% total) and natural albite (68.74 wt% SiO_2_, 19.44 wt% Al_2_O_3_, 11.75 wt% Na_2_O, 0.03 wt% K_2_O, 99.96 wt% total) from the in-house standard collection, which plot close to the stoichiometry line. An additional Smithsonian anorthite (ANO NMNH137041^41^;) was frequently analysed as secondary monitor during analysis of experimental samples. For the other analysed elements, K, Fe, Mg, Mn, Ti, Sr, and Ba, microcline (Smithsonian), synthetic fayalite, forsterite, pyrolusite, rutile, strontianite, and barium titanate (in-house) standards were used, respectively. Higher-order excitation lines were suppressed using energy windows in the single channel analyser. Standardisation was improved by repeated analysis of the main standards as unknowns.

Plagioclase crystals from natural samples were analysed with 15 kV acceleration voltage (to excite the Fe-K*α* line), 20 nA beam current, and 10 *μ*m beam diameter - conditions at which alkali migration is negligible during the analysis time even in albite-rich samples. The small size of plagioclase crystals from experiments precluded analysis with a large beam diameter, so that a focused beam was used and the acceleration voltage was reduced to 12 kV (to lower the excitation volume). Consequently, the beam current was decreased to ca. 4.5 nA. Suitable analysis conditions for plagioclase at the EPMA were determined by monitoring of count rates over time. The uncertainties were calculated from the relative count statistical error (r) based on the signal intensities of the standard compared to the samples, which was then translated into the uncertainty of the apfu.

Compositions of experimental glasses and pyroxenes were analysed with standard-based energy-dispersive X-ray spectroscopy (EDS) using a JEOL JSM-6390 LA scanning electron microscope (SEM), equipped with a Thermo Fisher NSS7 EDS system (30 mm^2^ silicon drift detector) and an in-column Faraday cup attached to an Ampere-meter, at the Department of Earth Sciences, ETH Zurich. Analysis conditions were: 15 kV acceleration voltage, ca. 2.3–2.4 nA beam current (recorded before each measurement), corresponding to ca. 20–25% dead time, and 30 s acquisition time. A ZAF matrix correction was applied to the analyses. Expected uncertainties of the measurements are in the range of a few percent for major elements.

### Availability of materials and data

All data generated during this study are included in this published article and its Supplementary Information file.

## Electronic supplementary material

Supplement A
